# Depression in late adolescence: a cross-sectional study in senior high schools in Greece

**DOI:** 10.1186/s12888-015-0584-9

**Published:** 2015-08-18

**Authors:** Konstantina Magklara, Stefanos Bellos, Dimitrios Niakas, Stelios Stylianidis, Gerasimos Kolaitis, Venetsanos Mavreas, Petros Skapinakis

**Affiliations:** 1 Department of Psychiatry, School of Medicine, University of Ioannina, Ioannina, 45110 Greece; 2 School of Social Sciences, Hellenic Open University, Patras, Greece; 3 Department of Psychology, Panteion University of Social and Political Sciences, Athens, Greece; 4 Department of Child Psychiatry, Athens University Medical School, Aghia Sophia Children’s Hospital, Athens, Greece

## Abstract

**Background:**

Depression is a common mental health problem in adolescents worldwide. The aim of the present study was to investigate the prevalence, comorbidity and sociodemographic and socioeconomic associations of depression and depressive symptoms, as well as the relevant health services use in a sample of adolescents in Greece.

**Methods:**

Five thousand six hundred fourteen adolescents aged 16–18 years old and attending 25 senior high schools were screened and a stratified random sample of 2,427 were selected for a detailed interview. Psychiatric morbidity was assessed with a fully structured psychiatric interview, the revised Clinical Interview Schedule (CIS-R). The use of substances, such as alcohol, nicotine and cannabis, and several sociodemographic and socioeconomic variables have been also assessed.

**Results:**

In our sample the prevalence rates were 5.67 % for the depressive episode according to ICD-10 and 17.43 % for a broader definition of depressive symptoms. 49.38 % of the adolescents with depressive episode had at least one comorbid anxiety disorder [OR: 7.76 (5.52-10.92)]. Only 17.08 % of the adolescents with depression have visited a doctor due to a psychological problem during the previous year. Anxiety disorders, substance use, female gender, older age, having one sibling, and divorce or separation of the parents were all associated with depression. In addition, the presence of financial difficulties in the family was significantly associated with an increased prevalence of both depression and depressive symptoms.

**Conclusions:**

Prevalence and comorbidity rates of depression among Greek adolescents are substantial. Only a small minority of depressed adolescents seek professional help. Significant associations with financial difficulties are reported.

**Electronic supplementary material:**

The online version of this article (doi:10.1186/s12888-015-0584-9) contains supplementary material, which is available to authorized users.

## Background

Depression is one of the leading causes of disease burden and disability across all age groups [1] and a major risk factor for suicide, substance abuse and serious social and educational impairments [2–4]. Although adolescents are often considered as a healthy population, they appear to be particularly vulnerable to depressive disorders [5]. Prevalence rates in childhood are low with no gender differences [6] and then increase significantly in adolescence, while gender differences emerge [7, 8]. Estimated 1-year prevalence rates of unipolar depression in mid to late adolescence range between 4–5 % and are comparable to those observed in the adult population [9, 10], while the cumulative probability of depression by the end of adolescence appears to be as high as 20 % [11, 12]. During the last decades the prevalence of depression in adolescence appears to have increased in the most recent birth cohorts [13]. Although it is not yet clear if this is due to a pure rise in the prevalence of the disorder or if it can be at least partially attributed to methodological problems, the World Health Organization reports a rise in the burden of depression globally and a World Health Assembly resolution in May 2012 called for a coordinated response to mental disorders at country level [14]. In Greece there has been limited research on the epidemiology of depressive disorders in adolescence. A recent study, which investigated depressive symptomatology in Greek adolescents attending senior high schools, reported a prevalence rate of 26.2 % [15]. An earlier study has also shown high prevalence of depressive symptoms in adolescents aged 12–17 years old [16]. A recent study in Greek adults reported that one month prevalence rate of major depression in the subgroup of youth under 24 years old increased considerably between the years 2008 and 2011. In the same study, younger age was identified as a risk factor for major depression in the Greek population [17]. However, it is worth noting that the above mentioned study was a telephone survey. Another study of Greek adults, which has implemented a different sampling procedure and has used a fully structured psychiatric interview, reported a lower prevalence rate of depression, which increased with age [18].

Depression in adolescence shows substantial comorbidity with anxiety and substance abuse disorders and this finding has been well established through various studies [19]. Comorbid disorders are more common than the “pure” types, while comorbidity between anxiety and depressive disorders in adolescents is greater than within the diagnostic group of anxiety disorders. Another common finding regarding adolescent depression is the relatively low use of health services, despite the high prevalence and disability associated with the disorder. Health services utilization appears to be even lower in cases of non-comorbid depression [20].

Regarding the correlates of depression, beyond the well established sociodemographic factors of age and gender, socioeconomic factors are also important. A socioeconomic gradient in adolescent depression has been documented in both the United States and Europe. Similarly to findings reported by studies in adults [21], lower socioeconomic status has been correlated with a greater prevalence of depression in adolescents [22].

The aim of the present study was to investigate the prevalence, comorbidity and sociodemographic/ socioeconomic associations of depressive disorders and depressive symptoms, as well as some aspects of the health services use, in late adolescents in Greece. Greece has recently entered a long period of economic crisis with fundamental adverse effects on many areas of the life of the population. Our study took place during the years 2007 and 2008 just before the eruption of the 2009 financial crisis. We therefore consider as important the investigation of some significant mental health aspects of Greek adolescents during the crucial period that preceded the onset of the current socioeconomic crisis.

## Methods

### Description of the data set

The data reported here are derived from the “Epirus School Project” [23] which is a cross-sectional survey carried out in selected upper secondary schools in Greece. The study was approved by the Ethical committee of the Ministry of Education and the Greek Educational Institute and was conducted according to the Helsinki declaration. The study was also approved by the Head of each participating School. All students in the selected schools were invited to participate in the study, while the participation was voluntary. Consent was actively obtained from both the students and their parents.

### Secondary education in Greece

Secondary education in Greece is distinguished into lower secondary (grades 7–9; ages 13–15 years; attendance is compulsory) and upper secondary (grades 10–12; ages 16–18 years; attendance is not compulsory). Upper secondary schools are further distinguished into senior high schools (Lyceum) and technical vocational schools with the majority of students (75 %) attending senior high schools. In the “Epirus School Project” only senior high schools were selected (age of pupils 16–18 years). At the time of the design of the study approximately 75,000 students attended 1,193 senior high schools in Greece.

### Sampling of schools and pupils

Schools were selected according to the following rules: a) all senior high schools of the major cities in the north-western part of Greece (regions of Epirus and Aetoloakarnania) due to the proximity of this area to the University of Ioannina, b) all senior high schools in one randomly selected district of the Athens Metropolitan Area (the district of Kallithea was selected), c) all senior high schools of the island of Paros in the Aegean Sea (the island was conveniently selected due to the close collaboration of the schools and mental health units of the island with the Panteion University). A total of 25 schools took part in the study. The mean number of participants per school was 267 pupils ranging from 100 to 502. The main fieldwork took place between January 2007 and April 2008.

### Design of the study and data collection procedure

The study used a two-phase design [24]. In the first phase, all consenting students (*N* = 5,614, response rate 82 %) were administered a brief screening instrument in the classroom. The screening instrument of the first phase was developed from the revised clinical interview schedule (CIS-R) used in the second phase of the study. Students were selected for the second phase psychiatric interview using a stratified random sampling procedure according to the scores on the screening questionnaire: 100 % of those scoring high on the screening instrument (>75th percentile), 30 % of those scoring in the middle and 10 % of those scoring low (<25th percentile). The second phase (*N* = 2,431, response rate 95 %) consisted of the computerized version of a fully-structured psychiatric interview (see next section) and was carried out in the computer laboratories of the schools. It is noted that in two schools (both in the island of Paros) all consenting students were interviewed (that is the two phases were merged into one). The reason was the availability of the fieldworkers of the island of Paros, which allowed us to provide the instrument of the CIS-R interview in full to all consenting students. From the remaining 1,960 pupils who were selected according to the stratified random sampling procedure, 926 (47.2 %) were on the 100 % stratum, 866 (44.2 %) on the 30 % stratum and 168 (8.6 %) on the 10 % stratum. Four out of the 2,431 selected pupils had missing values on the sociodemographic questions (administered in the first phase of the study) and therefore 2,427 pupils were used in the final analysis.

### Assessment of psychiatric morbidity: the revised clinical interview schedule (CIS-R)

Depressive and other psychiatric symptoms were assessed with the revised clinical interview schedule (CIS-R), a fully structured psychiatric interview designed to be used by trained lay interviewers [25]. The CIS-R was the main instrument used in the national psychiatric morbidity surveys in the UK [26] and has been used in several other similar surveys around the world. A computerized version has also been developed and found to be comparable with the regular interview [27]. The CIS-R was originally designed to assess symptoms in participants above 16 years old but has been previously used in teenagers above 14 years old in Australia [28]. The CIS-R assesses the presence and severity of 14 common psychiatric symptoms (psychosomatic symptoms, fatigue, concentration/memory problems, sleep problems, irritability, depressive mood, depressive ideas, general worry, worry about physical health, free-floating anxiety, phobias, panic anxiety, compulsions and obsessions). Two screening questions in each section ask about the presence of the symptom during the past month and then there is a more detailed assessment of the presence, frequency, duration and severity of the symptom during the past seven days. Based on the above-mentioned characteristics of the symptoms each one of the 14 symptoms is rated with an individual score on a scale ranging from 0 to 4 (except depressive ideas scored from 0 to 5). In the first phase of the study we used the screening questions of the several symptom sections of the CIS-R. The full interview was taken by those students selected for the second phase of the study. Additional questions enable the application of the International Classification of Diseases – 10th edition (ICD-10) research diagnostic criteria for common mental disorders (including depressive episode, phobic disorders, generalized anxiety disorder, panic disorder and obsessive-compulsive disorder) using specially developed computerized algorithms.

The Greek version of the CIS-R was translated and back-translated using the procedure recommended by the World Health Organization http://www.who.int/substance_abuse/research_tools/translation/en/index.html. The psychometric properties of the Greek version of the CIS-R including its factor structure and internal consistency have been reported by Skapinakis et al. 2011 [29]. A test-retest reliability of the CIS-R was carried out in a subset of the present data set (two schools of the city of Ioannina with an interval between assessments of 2 weeks) and was found to be 0.84 [23].

### Assessment of depressive episode and depressive symptoms

As mentioned above, there are two depression-related sections in the CIS-R: in the first section (“depressive mood) respondents are asked about feeling sad, miserable or depressed, or being unable to enjoy or take an interest in things. More detailed questions ask about the frequency and intensity of these symptoms. In the second section (“depressive ideas”), respondents are asked about feelings of guilt, inadequacy and hopelessness and whether they thought that life was not worth living. Additional questions throughout the remaining CIS-R sections enable the application of ICD-10 diagnostic criteria for depressive episode. For the purposes of the present paper we defined a single variable for depressive episode, which includes all severities of depressive episodes according to ICD-10 (mild, moderate, severe). The reason was that we expected very low prevalence rates of the more severe types of the disorder, since our sample were active pupils able to attend school. Apart from this formal definition of depression, in our analyses we have also used a broader definition of “depressive symptoms” irrespectively of meeting the ICD-10 diagnostic criteria for a depressive episode. This was defined as having a score of two or more (denoting presence of clinically significant symptoms) in both the “depressive mood” and “depressive ideas” sections of the CIS-R.

### Socioeconomic and sociodemographic variables

Information about several sociodemographic and socioeconomic variables was obtained from the students in the first phase of the study. The variables included: gender, own age, parent’s age, parent’s marital status, number of brothers and sisters, mother’s educational status, father’s educational status, mother’s employment status, and father’s employment status. The variable of the employment status of the mother included the additional category “looks after the house”, which was not included in the employment status of the father, since a considerable proportion of women, but not men, in Greece choose to stay out of the labour market, in order to look after the house. Students were also asked to subjectively rate their academic performance in school on a 4-point scale (excellent, very good, good, fair). In Greece, where typical 16–18 years-old adolescents have not yet entered the labour market, neither have they completed their education, own educational level or occupation cannot be used as a measure of personal social position. Academic performance in school has been often used in the literature as a measure of the social position of the pupils in school [30–32]. Further, adolescents were asked to rate their relationship with mother and father (excellent, very good, good, fair, bad). In addition we asked students to subjectively assess their family’s financial condition by asking them whether their family was having any financial difficulties. The specific question asked was: “How do you think that your family is doing financially?” The possible answers included: “My family experiences no financial difficulties”, “My family experiences very few financial difficulties”, “My family experiences some financial difficulties” and “My family experiences a lot of financial difficulties”.

### Other variables

We obtained information about the use of health services in the second phase of the study. The specific question asked was: “How many times did you consult a doctor (family doctor, pediatrician, hospital doctor or any other doctor) for any reason during the last 12 months?” The possible answers were: “None”, “1-2 times”, “3-4 times”, “5-6 times”, “7-10 times” and “More than 10 times”. A second similar question followed, asking about doctor consultations specifically for a psychological reason. Additionally, we have investigated the use of substances, such as alcohol, nicotine and cannabis. For the purposes of the present paper we have defined frequent alcohol use as the consumption of hard liquor at least once weekly, smoking as smoking cigarettes daily and cannabis use as having tried cannabis at least once in their life.

### Statistical analyses

The analyses were all conducted using the statistical software package STATA 12.0. To take into account the potential effect of clustering of our data (since adolescents were nested into 25 schools) we first carried out a two-level logistic model (level 1: individuals, level 2: schools) in Stata using the gllamm command [33]. We also performed the models with the survey commands of Stata using school as the stratum. Results were very similar with both models and therefore in the paper we present the results using the survey commands because their use is more widespread in the literature. It should be noted that the effect of schools was negligible with an intraclass correlation coefficient close to zero (<0.08). In all analyses we have used probability weights to take into account the stratified random sampling procedure.

The associations between health measures and sociodemographic and socioeconomic variables were investigated using logistic regression models. We used two dependent variables: (i) meeting the criteria for a depressive episode according to ICD-10 and (ii) experiencing substantial depressive symptoms (depressive mood and depressive ideas concurrently), irrespectively of meeting the criteria of a depressive episode. For each dependent variable we have initially calculated odds ratios adjusted only for age and gender and then odds ratios adjusted additionally for all other variables. Comorbidity was investigated using odds ratios calculated from logistic regression models, where the comorbid condition was the dependent variable and depression (either yes or no) the independent variable. Using similar models we investigated the use of health services. Frequent doctor visits was the dependent variable and was defined as having visited a doctor more than twice during the previous 12 months for any reason or at least once for a psychological reason. For the purposes of the latter analysis we have created a variable for depression with three values: “no depression”, “pure depression” (meeting the criteria for a depressive episode only) and “comorbid depression” (meeting the criteria for a depressive episode and for at least one anxiety disorder).

## Results

### Description of the sample

Overall 5,614 students took part in the first phase of the study (55 % girls, 41 % 10th grade, 31 % 11th grade, 28 % 12th grade), while 2,431 students were interviewed in the second phase (59 % girls, 39 % 10th grade, 32 % 11th grade, 29 % 12th grade). A detailed table of the sociodemographic characteristics of the whole sample in both phases of the study is given in Additional file 1: Table S1. Due to the stratified sampling procedure there were more female than male students in the second phase.

### Prevalence of depression

The prevalence of “depressive episode” according to the ICD-10 and “depressive symptoms” by gender are shown in Table 1. For all variables investigated prevalence was significantly higher among the girls in our sample (*p* < 0.001). Having concurrent depressive symptoms only (17.4 %, 95 % CI: 15.81-19.17) was three times more common than suffering a depressive episode according to the criteria of ICD-10 (5.7 %, 95 % CI: 4.90-6.56).

**Table 1:** Prevalence of depressive disorders in 2427 16–18 years-old adolescents in Greece, by gender

	Total	Female	Male
	N ^a^ (%) 95 % CI ^b^	N ^a^ (%) 95 % CI ^b^	N ^a^ (%) 95 % CI ^b^
Depressive episode according to ICD-10	246 (5.7 %)	194 (8.9 %)	52 (2.6 %)
	4.9 % - 6.6 %	7.5 % - 10.4 %	1.9 % - 3.5 %
Depressive symptoms^c^	643 (17.4 %),	472 (24 %)	171 (11 %)
	15.8 % - 19.2 %	21.6 % - 26.7 %	9 % - 13.3 %

^a^Actual number of observations; percentages are weighted to take into account the stratified random sampling procedure; ^b^ CI: Confidence Interval; ^c^Depressive symptoms: Experiencing depressive mood and depressive ideas but not meeting full criteria for ICD-10 depressive episode (see methods)

**Fig. 1:**
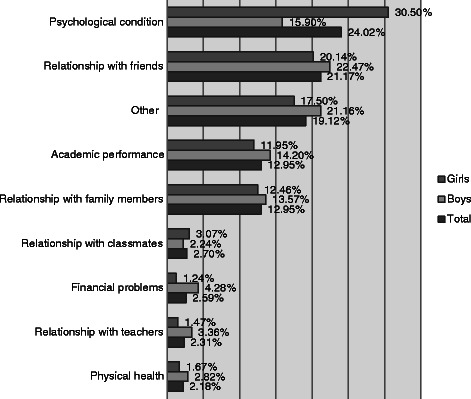
Main reasons for being sad or depressed in 16–18 years old adolescents in Greece

Figure 1 presents the reason reported by respondents about their depressed mood (“What sorts of things made you feel sad, miserable or depressed or unable to enjoy or take an interest in things in the past week?). The most common reasons were “my psychological condition” (24 %) and “problems with relationships with friends” (21.2 %), while the least common was “my physical health” (2.2 %).

### Co-morbidity

Table 2 presents the co-morbidity rates of depressive episode with main anxiety disorders and alcohol, smoking and cannabis use. 49.4 % of the adolescents with depressive episode had at least one comorbid anxiety disorder, while the respective frequency for adolescents without depression was 9.4 % [OR: 7.76 (5.52-10.92)]. All anxiety disorders were significantly more common among depressive adolescents. The strongest association was reported for generalized anxiety [OR: 7.92 (5.35-11.74)]. 24.5 % of the adolescents with depression reported consuming hard liquor at least once weekly [OR: 1.85 (1.27-2.70)], 22 % responded smoking cigarettes daily [OR: 2.05 (1.39-3.04)], while 10.3 % reported having used cannabis at least once [OR: 3.48 (2.05-5.93)].

**Table 2:** Comorbidity of depressive episode with other psychiatric disorders/ clinical conditions in 16–18 years-old adolescents in Greece

	% of Adolescents with depressive episode^a^	Odds Ratio^b^ (95 % CI^c^)
Comorbid condition:		
At least one anxiety disorder	49.4	7.76 (5.52-10.92)
*OCD* ^*d*^	17.1	5.32 (3.48-8.14)
*GAD* ^*e*^	27.6	7.92 (5.35-11.74)
*Panic disorder*	8.7	4.03 (2.14-7.57)
*Phobias*		
*Agoraphobia*	3.4	2.53 (1.01-6.30)
*All other phobias*	12.4	2.93 (1.83-4.68)
Alcohol^**f**^	24.5	1.85 (1.27-2.70)
Cigarette smoking^g^	22.1	2.05 (1.39-3.04)
Cannabis^h^	10.3	3.48 (2.05-5.93)

^a^All percentages are weighted to account for the stratified random sampling; ^b^CI: Confidence Interval; ^c^Odds ratios adjusted for age and sex and calculated from logistic regression models with the comorbid condition as the dependent variable and depressive episode (either yes or no) as the independent variable. The reference group for the reported odds ratios is “adolescents without depression” (e.g., the odds of at least one anxiety disorder was 7.76 times higher for participants with depression compared to participants without depression); ^d^OCD: Obsessive Compulsive Disorder; ^e^GAD: Generalized Anxiety Disorder; ^f^Alcohol use defined as consumption of hard liquor at least once weekly; ^g^Cigarette smoking defined as smoking cigarettes daily; ^h^Cannabis use defined as having tried cannabis at least once

### Use of health services

The use of health services is shown in Table 3. 10.4 % of the adolescents with “pure” depression (non-comorbid) and 23.9 % of those with comorbid depression had visited a doctor for a mental health reason at least once during the previous year. On the whole 17.1 % of the adolescents with a current depressive episode had seen a doctor for a psychological problem or symptom during the previous year. Statistically significant differences in frequent doctor visits were observed for adolescents who received at least two diagnoses and only for consultations due to psychological reasons [OR: 2.54 (1.55-4.19)].

**Table 3:** Health services use among 16–18 years-old adolescents attending senior high schools in Greece (*N* = 2427)

	Presence of ICD-10 Depressive Episode		
	*No Depression*	*"Pure" Depression*	*Comorbid Depression*
	*%* ^*a*^ *Odds Ratios* ^*b*^ *(95 % CI* ^*c*^ *)*	*%* ^*a*^ *Odds Ratios* ^*b*^ *(95 % CI* ^*c*^ *)*	*%* ^*a*^ *Odds Ratios* ^*b*^ *(95 % CI* ^*c*^ *)*
Frequent doctor visits ^d^			
*For any reason*	17.7 %	16.6 %	25.8 %
	1 (Reference Category)	0.91 (0.50-1.64)	1.59 (0.97-2.60)
*For a psychological reason*	11.3 %	10.4 %	23.9 %
	1 (Reference Category)	0.96 (0.52-1.79)	2.54 (1.55-4.19)

^a^All percentages are weighted to account for the stratified random sampling; ^b^Odds ratios adjusted for age and sex and calculated from logistic regression models with frequent doctor visits as the dependent variable and depressive episode (no depression,“ pure” or “comorbid” depression) as the independent variable (e.g., the odds of frequent doctor visits for a psychological reason was 1.95 times higher for participants with comorbid depression compared to participants without depression); ^c^CI: Confidence Interval;^d^Frequent doctor visits defined as having visited a doctor more than twice during the previous 12 months for any reason or at least once for a psychological reason

### Sociodemographic and socioeconomic correlations

Table 4 presents crude and adjusted odds ratios for depressive episode according to ICD-10 and our broader definition of depressive symptoms. After adjusting for all sociodemographic and socioeconomic indicators, female gender was statistically significantly associated with a higher risk for both dependent variables investigated in our analyses. The association was stronger for depressive episode [OR: 3.93 (2.65-5.82)]. Older age as expressed by the school grade of the pupils [OR: 1.69 (1.16-2.47)] and having one sibling [OR: 2.20 (1.17-4.13)] were both significantly associated with a greater risk for depressive episode according to ICD-10. Parent’s divorce or separation [OR: 2.02 (1.34-3.03)] was associated with an increased prevalence of depressive symptoms.

**Table 4:** – Logistic regression analyses for depressive episode and depressive symptoms in 16–18 years-old adolescents in Greece. Odds ratios of ICD-10 depressive episode and depressive symptoms adjusted for several socioeconomic status indicators in adolescents 16–18 years old attending senior high schools in Greece (*N* = 2427)

	Depressive episode (ICD-10)		Depressive symptoms	
	Crude OR^a^ (95 % CI^b^)	Adjusted OR^a^ (95 % CI^b^)	Crude OR^a^ (95 % CI^b^)	Adjusted O^a^ (95 % CI^b^)
Female gender	3.70 (2.55-5.36)	**3.93 (2.65-5.82)**	2.56 (1.98-3.31)	**2.55 (1.96-3.31)**
Grade				
10th	1.00	1.00	1.00	1.00
11th	1.23 (0.83-1.81)	1.22 (0.81-1.82)	0.85 (0.63-1.14)	0.87 (0.65-1.18)
12th	1.71 (1.16-2.48)	**1.69 (1.16-2.47)**	1.05 (0.80-1.39)	1.03 (0.78-1.37)
Parent’s marital status				
Married	1.00	1.00	1.00	1.00
Divorced/ Separated	1.83 (1.08-3.10)	1.69 (0.96-2.98)	2.13 (1.43-3.18)	**2.02 (1.34-3.03)**
Widow	0.89 (0.43-1.82)	0.98 (0.43-2.22)	1.10 (0.62-1.92)	1.29 (0.68-2.45)
Number of siblings				
None	1.00	1.00	1.00	1.00
One	1.73 (1.01-2.94)	**2.20 (1.17-4.13)**	1.10 (0.70-1.73)	1.25 (0.76-2.05)
Two	1.23 (0.67-2.26)	1.47 (0.74-2.95)	0.90 (0.56-1.45)	0.94 (0.56-1.60)
Three or more	1.03 (0.54-1.97)	1.19 (0.57-2.52)	1.17 (0.69-1.99)	1.17 (0.67-2.06)
Father’s educational level				
Primary	1.00	1.00	1.00	1.00
Secondary Basic	1.03 (0.56-1.85)	1.07 (0.59-1.94)	0.74 (0.48-1.14)	0.75 (0.47-1.20)
Secondary Complete	1.01 (0.63-1.62)	1.13 (0.67-1.90)	0.71 (0.49-1.03)	0.80 (0.53-1.20)
Technological degree	1.41 (0.81-2.45)	1.46 (0.80-2.65)	0.81 (0.52-1.26)	0.90 (0.56-1.45)
University degree	0.94 (0.57-1.54)	1.06 (0.59-1.92)	0.78 (0.54-1.14)	0.97 (0.60-1.55)
Mother’s educational level				
Primary	1.00	1.00	1.00	1.00
Secondary Basic	0.83 (0.47-1.46)	0.79 (0.44-1.40)	0.84 (0.54-1.33)	0.87 (0.53-1.41)
Secondary Complete	0.84 (0.53-1.31)	0.76 (0.45-1.30)	0.78 (0.54-1.13)	0.83 (0.55-1.27)
Technological degree	1.12 (0.62-2.02)	0.86 (0.43-1.72)	0.99 (0.61-1.60)	1.03 (0.60-1.78)
University degree	0.97 (0.59-1.60)	0.92 (0.46-1.83)	0.79 (0.53-1.18)	0.86 (0.52-1.43)
Father’s employment status				
Public sector employee	1.00	1.00	1.00	1.00
Private sector employee	1.29 (0.85-1.95)	1.29 (0.85-1.97)	1.27 (0.91-1.77)	1.29 (0.90-1.86)
Self-employed	0.92 (0.62-1.37)	0.98 (0.65-1.48)	1.08 (0.81-1.45)	1.13 (0.82-1.56)
Retired	0.41 (0.17-1.00)	**0.39 (0.16-0.99)**	1.50 (0.81-2.78)	1.55 (0.82-2.96)
Unemployed/ Other	1.02 (0.58-1.80)	0.95 (0.50-1.80)	1.25 (0.79-1.98)	1.01 (0.63-1.78)
Mother’s employment status				
Public sector employee	1.00	1.00	1.00	1.00
Private sector employee	0.95 (0.60-1.51)	0.82 (0.50-1.34)	0.93 (0.66-1.31)	0.80 (0.55-1.16)
Self-employed	0.83 (0.49-1.41)	0.84 (0.47-1.50)	0.91 (0.62-1.35)	0.90 (0.59-1.36)
Unemployed	1.16 (0.63-2.15)	1.07 (0.54-2.13)	1.30 (0.77-2.20)	1.03 (0.59-1.81)
Looks after house	0.74 (0.49-1.14)	0.76 (0.47-1.23)	0.91 (0.65-1.26)	0.89 (0.61-1.28)
Retired/ Other	0.78 (0.39-1.54)	0.73 (0.34-1.57)	0.99 (0.59-1.64)	0.87 (0.50-1.51)
Financial difficulties				
No	1.00	1.00	1.00	1.00
Few	1.26 (0.85-1.86)	1.19 (0.79-1.79)	1.32 (0.98-1.75)	1.28 (0.95-1.72)
Some/ A lot	2.33 (1.48-3.67)	**2.23 (1.40-3.55)**	2.44 (1.73-3.44)	**2.29 (1.60-3.28)**
School performance				
Excellent/ Very good/ Good	1.00	1.00	1.00	1.00
Fair	1.49 (1.03-2.16)	1.45 (0.97-2.18)	1.28 (0.96-1.70)	1.13 (0.82-1.54)

^a^OR: Odds ratio;^b^CI: Confidence Interval; Bold numbers indicate statistical significance

Regarding parent’s employment status, the retirement of the father was significantly associated with a lower risk for depressive episode [OR: 0.39 (0.16-0.99)]. Some or a lot financial difficulties in the family showed a statistically significant association with both dependent variables. School performance was not associated at a statistically significant level with our dependent variables. We have, however, performed separate analyses for the boys and girls in our sample and we found that lower academic performance increases the risk of depressive episode only for the boys of the sample [OR: 2.72 (1.28-5.78)].

## Discussion

### Main findings

5.7 % of the adolescents in our sample met the criteria of a depressive episode according to ICD-10. One in two adolescents with depression reported at least one comorbid anxiety disorder. Frequent doctor consultations due to psychological reasons were positively associated with depression. Less than one in five depressed adolescents had visited a doctor during the previous 12 months due to a mental health reason. A number of sociodemographic and socioeconomic factors were associated with adolescents’ depression. Among them, the presence of financial difficulties in the family, as perceived by the adolescent, was significantly associated with both depressive outcomes investigated in the present paper.

### Comparison with other studies

The prevalence rate of depression found in our study is similar to ones reported by other studies conducted in Europe and the Unites States. Costello et al. performed a large meta-analysis of approximately 60,000 children born over the past 30 years, from studies that had used a structured psychiatric interview to assess depression. For the subgroup of adolescents aged 13–18 years old they reported an overall prevalence estimate of 5.6 % [10]. Past studies in adolescent populations in Greece have not used structured psychiatric interviews and have mainly assessed depressive symptoms. Previous studies have chosen measures, such as the CES-D scale (center for epidemiological studies of depression scale) [15, 16] or the Delusions Symptoms States Inventory/states of Anxiety and Depression [34]. The most recent among the studies in Greek adolescents showed a prevalence rate of depressive symptomatology equal to 26.2 % [15], a figure which is somewhat higher than our estimate of 17.4 %. Studies conducted during the ‘90s on similar samples, found rates of depressive symptoms of as high as 33.4 % for males and 60.6 % for females [34] or even higher [16]. The observed differences could be explained through the different sampling frame and instruments used. Structured interviews, such as the CIS-R, may be more conservative in their estimate of symptoms compared to simpler scales [35].

Depression was significantly more common among the girls in our sample (*p* < 0.001).

Gender differences in the prevalence of depression among adolescents have been well established with approximately twice as many females than males reporting depressive disorders in mid-adolescence [7]. It is not yet clear whether the observed differences are real or emerge due to methodological issues [36]. Misclassification of questionnaires has been reported and it has been discussed that some items (like crying and lost interest in sex) are related in certain ways to female gender and, therefore, give gender-biased results in measuring depressiveness [37]. In the present study the assessment of depressiveness is based on a fully structured psychiatric interview and not only on a questionnaire. As a result, it is expected that the variation found may be less attributable to methodological artefacts.

Almost half of the adolescents with depression in the present study were presented with at least one comorbid anxiety disorder. The finding is consistent with figures reported by studies conducted in different populations. Comorbidity rates of as high as 75 % have been shown in some clinical samples [38, 39], with rates between 20 and 50 % more likely to be reported [40, 41]. An interesting consideration regarding comorbidity rates between depression and anxiety is the one stated by some researchers, who point out that studies may underestimate rates, since major depression accompanied by subclinical anxiety would not qualify as comorbidity [39]. The co-occurring anxiety, however, though subclinical at the moment, may have significant clinical implications later in development.

Alcohol, cigarette smoking and cannabis use were significantly more common among adolescents with depression in our study. The strong relationship between alcohol use and major depressive disorder in adolescents has been noted by many previous studies [42–44]. Theories about their etiological relation have proposed that depression increases the risk of alcohol dependence [45]. On the other hand, however, there is evidence that alcohol use disorders may not only exacerbate, but may also induce depression [46].

Cannabis use and depression are presented commonly as comorbid conditions in clinical and community populations [47, 48]. Nevertheless, the degree and the direction of their causal relation is a subject of controversy. Adolescents may use cannabis as a self-medication for their feelings of dysphoria, but cannabis use itself may significantly worsen, or even induce, such feelings [49, 50]. A number of cohort and well-designed cross-sectional studies have shown that it is the heavy and problematic cannabis use, rather than the infrequent one, which is associated with depression [51]. Our study, however, did not aim at exploring the effect of the frequency of cannabis use on depression.

Similarly, a number of epidemiological studies have investigated the association of cigarette smoking with adolescents’ mental health disorders [52, 53]. While externalizing disorders, such as conduct disorder or attention-deficit/hyperactivity disorder (ADHD) have been consistently related to adolescents’ cigarette smoking [54], findings about internalizing disorders, such as depression and anxiety, appear to be contradictory. Some studies report a significant relationship between these disorders and smoking [55, 56], while others have not confirmed any significant association [57, 58]. A recent study in Greek adolescents has shown that cigarette smoking was strongly associated with higher levels of emotional/ behavioural problems and the association was not moderated after controlling for the effects of possible covariates [59].

The present study presented evidence of socioeconomic inequalities in adolescent depression. On the whole, the association between socioeconomic position and depression across the lifespan remains a controversial area. Although lower socioeconomic status shows a robust association with high psychiatric morbidity, the results for depression are ambiguous [60]. In our study the socioeconomic risk factor was conceptualized through the financial difficulties of the family, as perceived and reported by the adolescent. Our finding is consistent with previous studies, which showed that adolescents, who thought that their socioeconomic status was somewhat or much worse off than their peers had a higher prevalence of depression [22]. In our study both depression according to ICD-10 and depressive symptoms correlated with financial difficulties of the family. Other studies, which investigated depressive symptomatology, have also reported associations with subjective measures of adolescent socioeconomic status [61]. A number of theories have tried to identify the pathways linking socioeconomic status and depression [62]. According to the stress theory lower socioeconomic status is associated on the one hand with higher levels of chronic stress due to financial difficulties, family problems and adverse living conditions, and with lower levels of personal resources, such as coping style, self-esteem, mastery and locus of control on the other. Many studies in depression are consistent with the above mentioned theory, while evidence supporting the strain theory, which underlines the decisive role of contextual and community features such as values, social welfare, social cohesion, infrastructure and policies, is conflicting [60]. Some researchers suggest that social determinants of health may be also explainable through the mechanism of status comparisons [63]. In our study we have not asked the adolescents about their family income. It is expected that one part of the information obtained through the question about the financial difficulties of the families of the adolescents may be related to aspects relevant to social comparisons. Likewise, the association in the boys of our sample of depression with lower academic performance, which could be seen as an indicator of the social position of the pupil in the school context, may also reflect a process of social comparisons among the adolescents.

Finally, the present study reports an association between depression and service utilization as expressed by frequent doctor consultations. The finding is consistent with previous studies suggesting that adolescents’ mental health problems increase help-seeking from all health-service providers [64]. In the Greek health system general practitioners do not act as a filter to specialized services and patients are not restricted to consult directly the health professional of their choice. As a result, medical doctors are usually the first professionals the patients seek, when in need. Less than one in five adolescents experiencing depression consulted a doctor during the last year due to their condition, while only one in ten depressed adolescents with no comorbid anxiety have seen a doctor. The finding is consistent with previous studies from Europe and the United States reporting that only a minority of adolescents with depression seeks professional help [20].

There are certain limitations of our study. The cross-sectional nature of our study should be taken into account when trying to interpret our results and draw causal inferences. Moreover, our sample included only pupils attending senior high schools (approximately 75 % of the school-attending adolescents of this age) and not those attending other school types (for example technical vocational schools). Additionally, in the present study we have included adolescents from urban areas. Our sample is not a typically representative sample of the Greek population and many areas were conveniently selected, however it represents a significant part of the population of the country, since it includes important aspects of the observed intranational geographical, economical and cultural diversity (urban mainland, metropolitan and island population). Furthermore, parental employment status was based on adolescents’ self-report, which may result in some misclassification. However, this kind of misclassification is expected to be random. Moreover, the question about parental employment status did not include information about the exact occupation and as a result an official “occupational status” classification was not possible.

Additionally, we have used a subjective socioeconomic variable, namely adolescents’ self-reports on the financial difficulties of their families. It has been suggested that directly questioning adolescents about their family’s income can be unreliable [31]. In the literature financial difficulties of the family have been often used as a socioeconomic indicator in studies investigating socioeconomic health inequalities in populations of children and adults [23, 65]. These studies have shown that subjective indicators may be equally or even more important compared to more objective indicators of socio-economic status [65]. Further, regarding the service utilization, help-seeking can be only reported in retrospect, and symptoms of depression only at present. As a result, help-seeking may be reported for psychological problems in adolescents without current symptoms and vice versa.

## Conclusions

During the last few years Greece is confronted with a serious economic crisis. Our study has tried to investigate some important aspects of adolescent depression in the country with data obtained just before the outbreak of the crisis. The present study reports a significant burden of depression for Greek adolescents, even before the crisis and its effects became evident, and suggests that there is an important association between depressive symptomatology and financial problems. Only a small proportion of the adolescents experiencing depression have used professional help. It could be expected that a socioeconomic crisis would further worsen the above picture. Further research, however, is needed to support this argument, with the goal to make the best possible use and distribution of available resources.

## Additional File

Additional File 1: **Basic description of the sample in the two phases of the study.** Table S1: Sociodemographic characteristics of the whole sample in Phase 1 (*n* = 5614) and Phase 2 (*N* = 2431) of the study. (DOCX 14 kb).

## Abbreviations

ICD-10: International Classification of Diseases – 10th edition
CIS-R: Clinical Interview Schedule Revised
